# Tris(2,4-di-*tert*-butyl­phen­yl) phosphate

**DOI:** 10.1107/S1600536810029673

**Published:** 2010-08-04

**Authors:** T. Vinuchakkaravarthy, C. K. Sangeetha, D. Velmurugan

**Affiliations:** a Centre of Advanced Study in Crystallography and Biophysics, University of Madras, Guindy Campus, Chennai 600 025, India

## Abstract

The title compound, C_42_H_63_O_4_P, was isolated from the leaves of *Vitex negundo*. Two of the *tert*-butyl groups are disordered over two orientations with occupancy ratios of 0.57 (1):0.43 (1) and 0.67 (1):0.33 (1). Several intra­molecular C—H⋯O inter­actions are observed in the mol­ecular structure.

## Related literature

For general background and the biological activity of *Vitex negundo*, see: Aswar *et al.* (2009 ▶); Chadha (1976 ▶); Kulkarni *et al.* (2008 ▶); Sahare *et al.* (2008 ▶); Rastogi *et al.* (2009 ▶). For the geometry of the 2,4-di-tert-butyl­phenyl group, see: Janse van Rensburg *et al.* (2006 ▶).
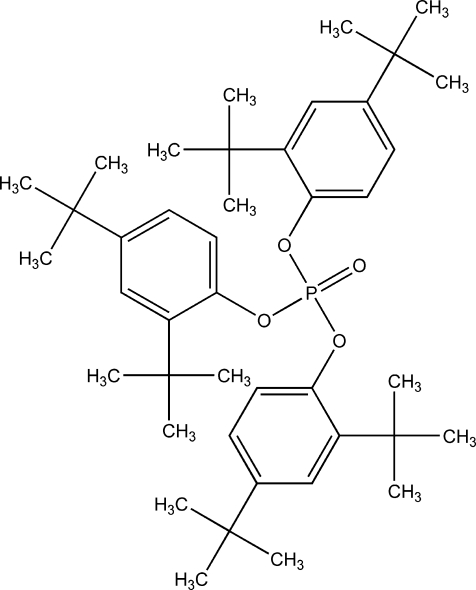


## Experimental

### 

#### Crystal data

C_42_H_63_O_4_P
*M* *_r_* = 662.89
Monoclinic, 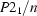

*a* = 15.702 (4) Å
*b* = 16.262 (4) Å
*c* = 16.262 (4) Å
β = 91.578 (6)°
*V* = 4150.9 (18) Å^3^
*Z* = 4
Mo *K*α radiation
μ = 0.10 mm^−1^
*T* = 293 K
0.25 × 0.22 × 0.19 mm

#### Data collection

Bruker Kappa APEXII CCD area-detector diffractometer
Absorption correction: multi-scan (*SADABS*; Sheldrick, 2008*a* ▶) *T*_min_ = 0.975, *T*_max_ = 0.981
40376 measured reflections
10329 independent reflections
5669 reflections with *I* > 2σ(*I*)
*R*_int_ = 0.048

#### Refinement

*R*[*F* ^2^ > 2σ(*F* ^2^)] = 0.057
*wR*(*F* ^2^) = 0.184
*S* = 1.02
10329 reflections
480 parameters
96 restraints
H-atom parameters constrained
Δρ_max_ = 0.37 e Å^−3^
Δρ_min_ = −0.34 e Å^−3^

### 

Data collection: *APEX2* (Bruker, 2004 ▶); cell refinement: *SAINT* (Bruker, 2004 ▶); data reduction: *SAINT*; program(s) used to solve structure: *SHELXS97* (Sheldrick, 2008*b* ▶); program(s) used to refine structure: *SHELXL97* (Sheldrick, 2008*b* ▶); molecular graphics: *ORTEP-3* (Farrugia, 1997 ▶); software used to prepare material for publication: *SHELXL97* and *PLATON* (Spek, 2009 ▶).

## Supplementary Material

Crystal structure: contains datablocks global, I. DOI: 10.1107/S1600536810029673/ci5107sup1.cif

Structure factors: contains datablocks I. DOI: 10.1107/S1600536810029673/ci5107Isup2.hkl

Additional supplementary materials:  crystallographic information; 3D view; checkCIF report

## Figures and Tables

**Table 1:** Hydrogen-bond geometry (Å, °)

*D*—H⋯*A*	*D*—H	H⋯*A*	*D*⋯*A*	*D*—H⋯*A*
C3—H3⋯O2	0.93	2.32	3.017 (3)	132
C12—H12*C*⋯O1	0.96	2.36	3.022 (4)	125
C13—H13*A*⋯O1	0.96	2.37	2.996 (3)	122
C16—H16⋯O2	0.93	2.38	3.023 (3)	126
C27—H27*B*⋯O3	0.96	2.40	3.032 (3)	123
C28—H28*B*⋯O3	0.96	2.33	2.990 (4)	125
C30—H30⋯O2	0.93	2.32	3.019 (3)	132
C36—H36*A*⋯O4	0.96	2.31	2.969 (3)	125
C37—H37*C*⋯O4	0.96	2.40	3.044 (3)	124

**Fig. 1.**
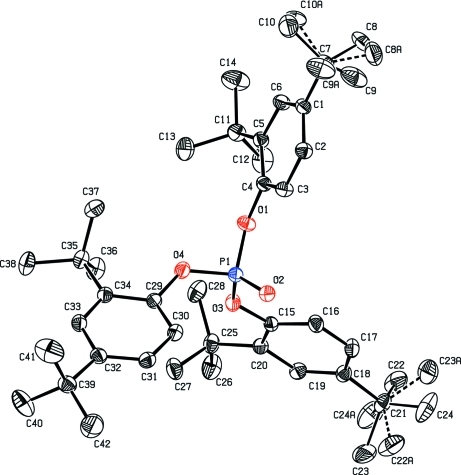
The molecular structure of the title compound, showing the atom-numbering scheme. Displacement ellipsoids drawn at the 30% probability level. Both disordercomponents are shown. H atoms have been omitted for clarity.

## supplementary crystallographic information

### Comment

*Vitex negundo* is one of the most common Indian medicinal plants which is used in Indian Folk medicine in the treatment of various ailments (Aswar *et al.*, 2009). Though, almost all parts of *V. negundo* are used, the leaves and the barks are the most important in the field of medicine (Chadha, 1976). The roots of *V. negundo* was reported to have antifilarial activity (Sahare *et al.*, 2008) and antihelmintic activity (Rastogi *et al.*, 2009). The methanol extract of *V. negundo* leaves standardized in terms of total polyphenol content was reported to have good free radical scavenging activity and anti-inflammatory activity (Kulkarni *et al.*, 2008). Many phenolic and polyphenolic compounds (secondary metabolites) were isolated from various plant sources so far, but the title compound is a new class of phenolic compound that has been isolated from the leaves of *V. negundo*, leading to an idea of new biosynthetic pathway of phenolics in plants and screening of their various biological activities for therapeutical approaches. It is a high molecular weight substituted phenolic compound. It is a class of primary anti-oxidant (free radical scavengers) which combines with peroxide radicals and breaks autocatalytic cycle. We report here its crystal structure.

In the title molecule (Fig. 1), the O—P—O angles around the P atom deviate significantly from ideal tetrahedral values. The dihedral angles between the benzene rings C1–C6 (A), C15–C20 (B) and C29–C34 (C) are: A/B = 73.0 (1)°, A/C = 67.0 (1)° and B/C = 76.9 (1)°. The O atoms are coplanar with the attached benzene rings which is evident from the torsion angles 175.6 (2)° (O1—C4—C5—C6), -174.4 (2)° (O3—C15—C16—C17) and -176.6 (2)° (O4—C29—C30—C31). In the molecular structure, several C—H···O interactions are observed (Table 1).

### Experimental

*Vitex negundo* leaves were collected in Kolli Hills, Namakkal, during April, 2008 and were identified by a botanical expert. Tris-(2,4-di-*tert*-butylphenyl)phosphate was isolated from the ethyl acetate extract of the leaves of *V. negundo* by silica gel column chromatography with gradient mixtures of hexane and ethyl acetate. White crystals were obtained by slow evaporation of an ethyl acetate solution. (m.p. 454 K-459 K).

### Refinement

The C7 and C21 *tert*-butyl groups are disordered over two orientations, with occupancies of 0.567 (14) and 0.433 (14), and 0.668 (7) and 0.332 (7), respectively. The corresponding bond distances involving the disorderded atoms were restrained to be equal. The U^ij^ components of the disordered atoms were approximated to isotropic behaviour. H atoms were positioned geometrically and allowed to ride on their parent C atoms, with C–H distances in the range 0.93–0.97 Å and with *U*_iso_(H) = 1.5*U*_eq_(C) for methyl H and 1.2*U*_eq_(C) for other H atoms.

### Crystal data

**Table:**

C_42_H_63_O_4_P	*F*(000) = 1448
*M**_r_* = 662.89	*D*_x_ = 1.061 Mg m^−^^3^
Monoclinic, *P*2_1_/*n*	Mo *K*α radiation, λ = 0.71073 Å
Hall symbol: -P 2yn	Cell parameters from 3749 reflections
*a* = 15.702 (4) Å	θ = 1.8–28.6°
*b* = 16.262 (4) Å	µ = 0.10 mm^−^^1^
*c* = 16.262 (4) Å	*T* = 293 K
β = 91.578 (6)°	Block, white
*V* = 4150.9 (18) Å^3^	0.25 × 0.22 × 0.19 mm
*Z* = 4	

### Data collection

**Table:**

Bruker Kappa APEXII CCD area-detector diffractometer	10329 independent reflections
Radiation source: fine-focus sealed tube	5669 reflections with *I* > 2σ(*I*)
graphite	*R*_int_ = 0.048
ω and φ scans	θ_max_ = 28.6°, θ_min_ = 1.8°
Absorption correction: multi-scan (*SADABS*; Sheldrick, 2008*a*)	*h* = −20→20
*T*_min_ = 0.975, *T*_max_ = 0.981	*k* = −21→21
40376 measured reflections	*l* = −19→21

### Refinement

**Table:**

Refinement on *F*^2^	Primary atom site location: structure-invariant direct methods
Least-squares matrix: full	Secondary atom site location: difference Fourier map
*R*[*F*^2^ > 2σ(*F*^2^)] = 0.057	Hydrogen site location: inferred from neighbouring sites
*wR*(*F*^2^) = 0.184	H-atom parameters constrained
*S* = 1.02	*w* = 1/[σ^2^(*F*_o_^2^) + (0.0826*P*)^2^ + 0.8971*P*] where *P* = (*F*_o_^2^ + 2*F*_c_^2^)/3
10329 reflections	(Δ/σ)_max_ = 0.001
480 parameters	Δρ_max_ = 0.37 e Å^−^^3^
96 restraints	Δρ_min_ = −0.34 e Å^−^^3^

### Special details

**Table:**

Geometry. All e.s.d.'s (except the e.s.d. in the dihedral angle between two l.s. planes) are estimated using the full covariance matrix. The cell e.s.d.'s are taken into account individually in the estimation of e.s.d.'s in distances, angles and torsion angles; correlations between e.s.d.'s in cell parameters are only used when they are defined by crystal symmetry. An approximate (isotropic) treatment of cell e.s.d.'s is used for estimating e.s.d.'s involving l.s. planes.
Refinement. Refinement of *F*^2^ against ALL reflections. The weighted *R*-factor *wR* and goodness of fit *S* are based on *F*^2^, conventional *R*-factors *R* are based on *F*, with *F* set to zero for negative *F*^2^. The threshold expression of *F*^2^ > σ(*F*^2^) is used only for calculating *R*-factors(gt) *etc*. and is not relevant to the choice of reflections for refinement. *R*-factors based on *F*^2^ are statistically about twice as large as those based on *F*, and *R*- factors based on ALL data will be even larger.

### Fractional atomic coordinates and isotropic or equivalent isotropic displacement parameters (Å^2^)

**Table:**

	*x*	*y*	*z*	*U*_iso_*/*U*_eq_	Occ. (<1)
P1	0.80834 (3)	0.31741 (3)	0.17910 (4)	0.04635 (17)	
O1	0.77840 (9)	0.32898 (10)	0.08683 (10)	0.0541 (4)	
O2	0.75319 (10)	0.26971 (9)	0.23155 (10)	0.0544 (4)	
O3	0.90074 (9)	0.28306 (9)	0.16739 (11)	0.0561 (4)	
O4	0.82627 (10)	0.40839 (9)	0.20586 (10)	0.0548 (4)	
C1	0.53071 (13)	0.35143 (13)	−0.01505 (15)	0.0488 (5)	
C2	0.54388 (14)	0.32741 (14)	0.06516 (15)	0.0533 (6)	
H2	0.4972	0.3147	0.0968	0.064*	
C3	0.62501 (14)	0.32170 (14)	0.10006 (15)	0.0522 (6)	
H3	0.6325	0.3057	0.1547	0.063*	
C4	0.69482 (13)	0.33985 (13)	0.05346 (14)	0.0450 (5)	
C5	0.68692 (14)	0.36596 (13)	−0.02816 (14)	0.0493 (5)	
C6	0.60322 (14)	0.36998 (14)	−0.05986 (15)	0.0543 (6)	
H6	0.5952	0.3861	−0.1144	0.065*	
C7	0.44112 (15)	0.35747 (16)	−0.05561 (16)	0.0607 (6)	
C8	0.4393 (6)	0.3024 (6)	−0.1327 (6)	0.085 (3)	0.567 (14)
H8A	0.4402	0.2457	−0.1163	0.102*	0.567 (14)
H8B	0.4882	0.3139	−0.1649	0.102*	0.567 (14)
H8C	0.3883	0.3132	−0.1648	0.102*	0.567 (14)
C9	0.3724 (4)	0.3209 (9)	0.0000 (5)	0.107 (3)	0.567 (14)
H9A	0.3950	0.2733	0.0279	0.129*	0.567 (14)
H9B	0.3235	0.3054	−0.0332	0.129*	0.567 (14)
H9C	0.3562	0.3612	0.0397	0.129*	0.567 (14)
C10	0.4207 (7)	0.4443 (5)	−0.0816 (8)	0.103 (3)	0.567 (14)
H10A	0.3932	0.4723	−0.0376	0.124*	0.567 (14)
H10B	0.3835	0.4434	−0.1294	0.124*	0.567 (14)
H10C	0.4724	0.4725	−0.0942	0.124*	0.567 (14)
C8A	0.4184 (10)	0.2826 (7)	−0.1025 (11)	0.108 (5)	0.433 (14)
H8AA	0.4199	0.2360	−0.0663	0.130*	0.433 (14)
H8AB	0.4583	0.2746	−0.1454	0.130*	0.433 (14)
H8AC	0.3621	0.2885	−0.1263	0.130*	0.433 (14)
C9A	0.3777 (6)	0.3814 (12)	0.0089 (6)	0.108 (4)	0.433 (14)
H9AA	0.3743	0.3383	0.0491	0.129*	0.433 (14)
H9AB	0.3227	0.3897	−0.0169	0.129*	0.433 (14)
H9AC	0.3961	0.4313	0.0355	0.129*	0.433 (14)
C10A	0.4376 (8)	0.4308 (8)	−0.1153 (8)	0.089 (4)	0.433 (14)
H10D	0.4383	0.4812	−0.0846	0.107*	0.433 (14)
H10E	0.3863	0.4279	−0.1487	0.107*	0.433 (14)
H10F	0.4861	0.4291	−0.1500	0.107*	0.433 (14)
C11	0.76410 (15)	0.38937 (16)	−0.08056 (16)	0.0599 (6)	
C12	0.8214 (2)	0.31473 (19)	−0.0926 (2)	0.0890 (10)	
H12A	0.8709	0.3310	−0.1219	0.107*	
H12B	0.7907	0.2735	−0.1236	0.107*	
H12C	0.8385	0.2927	−0.0399	0.107*	
C13	0.81492 (18)	0.45868 (17)	−0.0382 (2)	0.0806 (9)	
H13A	0.8394	0.4390	0.0128	0.097*	
H13B	0.7777	0.5041	−0.0277	0.097*	
H13C	0.8595	0.4764	−0.0733	0.097*	
C14	0.7358 (2)	0.4221 (3)	−0.1651 (2)	0.1123 (13)	
H14A	0.7848	0.4395	−0.1945	0.135*	
H14B	0.6981	0.4680	−0.1584	0.135*	
H14C	0.7067	0.3794	−0.1954	0.135*	
C15	0.92470 (13)	0.19949 (12)	0.15720 (14)	0.0466 (5)	
C16	0.86524 (15)	0.13759 (14)	0.16109 (16)	0.0573 (6)	
H16	0.8079	0.1506	0.1657	0.069*	
C17	0.89021 (16)	0.05613 (14)	0.15820 (16)	0.0589 (6)	
H17	0.8492	0.0149	0.1601	0.071*	
C18	0.97496 (15)	0.03491 (13)	0.15252 (14)	0.0525 (6)	
C19	1.03290 (15)	0.09966 (13)	0.14503 (14)	0.0524 (6)	
H19	1.0900	0.0863	0.1392	0.063*	
C20	1.01107 (14)	0.18317 (13)	0.14575 (14)	0.0479 (5)	
C21	1.00460 (17)	−0.05547 (14)	0.15407 (17)	0.0639 (7)	
C22	1.0388 (5)	−0.0797 (3)	0.0717 (3)	0.098 (2)	0.668 (7)
H22A	1.0961	−0.0601	0.0675	0.118*	0.668 (7)
H22B	1.0038	−0.0560	0.0286	0.118*	0.668 (7)
H22C	1.0381	−0.1386	0.0667	0.118*	0.668 (7)
C23	1.0779 (5)	−0.0663 (3)	0.2184 (4)	0.117 (3)	0.668 (7)
H23A	1.0990	−0.0133	0.2349	0.140*	0.668 (7)
H23B	1.1230	−0.0975	0.1947	0.140*	0.668 (7)
H23C	1.0572	−0.0949	0.2655	0.140*	0.668 (7)
C24	0.9340 (4)	−0.1140 (3)	0.1769 (6)	0.130 (3)	0.668 (7)
H24A	0.9142	−0.1003	0.2305	0.156*	0.668 (7)
H24B	0.9553	−0.1694	0.1774	0.156*	0.668 (7)
H24C	0.8877	−0.1096	0.1373	0.156*	0.668 (7)
C22A	0.9980 (12)	−0.0870 (8)	0.2385 (7)	0.115 (5)	0.332 (7)
H22D	1.0372	−0.0579	0.2743	0.138*	0.332 (7)
H22E	1.0115	−0.1446	0.2394	0.138*	0.332 (7)
H22F	0.9410	−0.0790	0.2569	0.138*	0.332 (7)
C23A	0.9422 (8)	−0.1038 (6)	0.0956 (9)	0.114 (5)	0.332 (7)
H23D	0.9081	−0.0656	0.0639	0.136*	0.332 (7)
H23E	0.9059	−0.1379	0.1276	0.136*	0.332 (7)
H23F	0.9743	−0.1375	0.0592	0.136*	0.332 (7)
C24A	1.0915 (9)	−0.0643 (8)	0.1216 (12)	0.128 (5)	0.332 (7)
H24D	1.1312	−0.0342	0.1559	0.154*	0.332 (7)
H24E	1.0923	−0.0432	0.0666	0.154*	0.332 (7)
H24F	1.1071	−0.1214	0.1215	0.154*	0.332 (7)
C25	1.07798 (15)	0.25155 (14)	0.13433 (17)	0.0602 (7)	
C26	1.16553 (17)	0.21537 (19)	0.1140 (2)	0.0894 (10)	
H26A	1.1867	0.1833	0.1597	0.107*	
H26B	1.2046	0.2593	0.1034	0.107*	
H26C	1.1599	0.1810	0.0661	0.107*	
C27	1.08814 (17)	0.30289 (17)	0.2130 (2)	0.0766 (8)	
H27A	1.1044	0.2677	0.2582	0.092*	
H27B	1.0350	0.3292	0.2244	0.092*	
H27C	1.1313	0.3439	0.2058	0.092*	
C28	1.0509 (2)	0.30698 (17)	0.0608 (2)	0.0821 (9)	
H28A	1.0922	0.3499	0.0544	0.099*	
H28B	0.9962	0.3309	0.0708	0.099*	
H28C	1.0473	0.2745	0.0115	0.099*	
C29	0.83683 (13)	0.44192 (12)	0.28703 (14)	0.0461 (5)	
C30	0.80912 (15)	0.39926 (14)	0.35457 (15)	0.0566 (6)	
H30	0.7860	0.3469	0.3482	0.068*	
C31	0.81596 (16)	0.43484 (14)	0.43200 (16)	0.0569 (6)	
H31	0.7980	0.4054	0.4774	0.068*	
C32	0.84884 (14)	0.51314 (14)	0.44336 (15)	0.0523 (6)	
C33	0.87591 (14)	0.55355 (14)	0.37267 (15)	0.0524 (6)	
H33	0.8981	0.6062	0.3791	0.063*	
C34	0.87219 (13)	0.52098 (12)	0.29329 (14)	0.0470 (5)	
C35	0.90342 (15)	0.56929 (13)	0.21791 (16)	0.0562 (6)	
C36	0.97073 (17)	0.51997 (16)	0.17182 (19)	0.0753 (8)	
H36A	0.9463	0.4690	0.1528	0.090*	
H36B	1.0185	0.5088	0.2083	0.090*	
H36C	0.9894	0.5513	0.1256	0.090*	
C37	0.82689 (18)	0.58997 (16)	0.16050 (17)	0.0707 (7)	
H37A	0.8464	0.6184	0.1129	0.085*	
H37B	0.7877	0.6243	0.1891	0.085*	
H37C	0.7987	0.5401	0.1437	0.085*	
C38	0.9459 (2)	0.65100 (16)	0.2443 (2)	0.0808 (9)	
H38A	0.9644	0.6798	0.1964	0.097*	
H38B	0.9941	0.6398	0.2801	0.097*	
H38C	0.9056	0.6842	0.2727	0.097*	
C39	0.85283 (17)	0.55513 (16)	0.52831 (16)	0.0635 (7)	
C40	0.9374 (2)	0.6003 (3)	0.5429 (2)	0.1156 (13)	
H40A	0.9396	0.6226	0.5976	0.139*	
H40B	0.9419	0.6441	0.5037	0.139*	
H40C	0.9837	0.5625	0.5365	0.139*	
C41	0.7845 (2)	0.6210 (3)	0.5294 (2)	0.1212 (15)	
H41A	0.7293	0.5956	0.5258	0.145*	
H41B	0.7910	0.6572	0.4834	0.145*	
H41C	0.7898	0.6519	0.5796	0.145*	
C42	0.8437 (3)	0.4962 (2)	0.5980 (2)	0.1315 (16)	
H42A	0.7873	0.4734	0.5963	0.158*	
H42B	0.8533	0.5246	0.6492	0.158*	
H42C	0.8847	0.4528	0.5933	0.158*	

### Atomic displacement parameters (Å^2^)

**Table:**

	*U*^11^	*U*^22^	*U*^33^	*U*^12^	*U*^13^	*U*^23^
P1	0.0426 (3)	0.0424 (3)	0.0536 (4)	0.0009 (2)	−0.0056 (2)	0.0007 (3)
O1	0.0401 (8)	0.0708 (10)	0.0512 (10)	0.0022 (7)	−0.0023 (7)	0.0024 (8)
O2	0.0534 (9)	0.0509 (8)	0.0583 (11)	−0.0046 (7)	−0.0054 (7)	0.0050 (7)
O3	0.0408 (8)	0.0416 (8)	0.0853 (12)	0.0017 (6)	−0.0073 (7)	−0.0025 (8)
O4	0.0687 (10)	0.0426 (8)	0.0530 (10)	−0.0014 (7)	0.0015 (8)	−0.0003 (7)
C1	0.0449 (12)	0.0515 (12)	0.0497 (15)	−0.0002 (10)	−0.0052 (10)	−0.0044 (10)
C2	0.0424 (12)	0.0627 (14)	0.0550 (16)	−0.0018 (10)	0.0024 (10)	0.0029 (11)
C3	0.0459 (12)	0.0635 (14)	0.0470 (14)	0.0016 (10)	−0.0003 (10)	0.0058 (11)
C4	0.0402 (11)	0.0466 (11)	0.0481 (14)	0.0005 (9)	−0.0034 (9)	−0.0028 (10)
C5	0.0484 (12)	0.0510 (12)	0.0484 (15)	−0.0038 (10)	0.0012 (10)	−0.0005 (10)
C6	0.0534 (13)	0.0635 (14)	0.0455 (15)	−0.0032 (11)	−0.0058 (10)	0.0022 (11)
C7	0.0475 (13)	0.0712 (16)	0.0624 (17)	−0.0014 (11)	−0.0130 (11)	−0.0046 (13)
C8	0.063 (4)	0.103 (6)	0.086 (5)	−0.008 (3)	−0.029 (3)	−0.020 (4)
C9	0.051 (3)	0.177 (8)	0.092 (5)	−0.024 (4)	−0.012 (3)	0.016 (5)
C10	0.077 (5)	0.067 (4)	0.163 (8)	0.015 (3)	−0.036 (5)	−0.014 (5)
C8A	0.088 (7)	0.074 (5)	0.161 (10)	−0.010 (5)	−0.042 (7)	−0.019 (6)
C9A	0.047 (4)	0.167 (9)	0.109 (6)	0.027 (6)	−0.009 (4)	−0.002 (6)
C10A	0.061 (5)	0.096 (7)	0.109 (7)	0.016 (4)	−0.037 (5)	0.012 (6)
C11	0.0535 (13)	0.0757 (16)	0.0509 (16)	−0.0086 (12)	0.0065 (11)	0.0057 (12)
C12	0.084 (2)	0.091 (2)	0.094 (2)	−0.0067 (17)	0.0424 (18)	−0.0130 (17)
C13	0.0713 (18)	0.0772 (18)	0.094 (2)	−0.0210 (15)	0.0206 (16)	0.0024 (16)
C14	0.087 (2)	0.185 (4)	0.065 (2)	−0.025 (2)	0.0074 (17)	0.040 (2)
C15	0.0464 (12)	0.0394 (10)	0.0536 (14)	0.0013 (9)	−0.0089 (10)	−0.0027 (9)
C16	0.0442 (12)	0.0505 (13)	0.0766 (18)	−0.0010 (10)	−0.0080 (11)	−0.0065 (12)
C17	0.0619 (15)	0.0447 (12)	0.0702 (18)	−0.0076 (11)	0.0007 (12)	−0.0071 (11)
C18	0.0661 (15)	0.0438 (11)	0.0477 (15)	0.0012 (10)	0.0044 (11)	−0.0045 (10)
C19	0.0523 (13)	0.0500 (12)	0.0552 (15)	0.0089 (10)	0.0056 (10)	−0.0016 (11)
C20	0.0495 (12)	0.0464 (11)	0.0478 (14)	0.0005 (10)	0.0010 (10)	−0.0014 (10)
C21	0.0820 (18)	0.0429 (12)	0.0674 (18)	0.0071 (12)	0.0121 (14)	−0.0018 (12)
C22	0.154 (5)	0.060 (3)	0.082 (4)	0.037 (3)	0.010 (3)	−0.013 (2)
C23	0.159 (6)	0.068 (3)	0.122 (5)	0.045 (3)	−0.043 (4)	0.000 (3)
C24	0.126 (5)	0.055 (3)	0.211 (8)	0.000 (3)	0.045 (5)	0.034 (4)
C22A	0.157 (9)	0.086 (6)	0.103 (8)	0.036 (7)	0.008 (7)	0.026 (6)
C23A	0.142 (9)	0.061 (5)	0.136 (9)	0.021 (6)	−0.021 (7)	−0.029 (6)
C24A	0.146 (9)	0.082 (6)	0.160 (10)	0.041 (6)	0.048 (7)	0.014 (7)
C25	0.0506 (13)	0.0508 (12)	0.0797 (19)	−0.0050 (10)	0.0095 (12)	−0.0014 (12)
C26	0.0595 (17)	0.0759 (18)	0.134 (3)	−0.0055 (14)	0.0286 (18)	0.0014 (18)
C27	0.0587 (16)	0.0699 (16)	0.101 (2)	−0.0147 (13)	−0.0051 (15)	−0.0120 (16)
C28	0.090 (2)	0.0639 (16)	0.094 (2)	−0.0081 (15)	0.0217 (17)	0.0146 (15)
C29	0.0455 (11)	0.0422 (11)	0.0504 (14)	0.0043 (9)	−0.0021 (9)	−0.0021 (10)
C30	0.0662 (15)	0.0452 (12)	0.0583 (17)	−0.0046 (11)	−0.0019 (12)	0.0020 (11)
C31	0.0661 (15)	0.0532 (13)	0.0512 (16)	−0.0006 (11)	0.0000 (11)	0.0060 (11)
C32	0.0469 (12)	0.0512 (12)	0.0584 (16)	0.0073 (10)	−0.0053 (10)	−0.0033 (11)
C33	0.0476 (12)	0.0447 (11)	0.0646 (17)	−0.0004 (9)	−0.0014 (11)	−0.0043 (11)
C34	0.0404 (11)	0.0435 (11)	0.0570 (15)	0.0035 (9)	0.0000 (9)	0.0001 (10)
C35	0.0597 (14)	0.0440 (11)	0.0653 (17)	−0.0014 (10)	0.0105 (12)	−0.0002 (11)
C36	0.0670 (16)	0.0689 (16)	0.091 (2)	−0.0023 (13)	0.0262 (15)	−0.0037 (15)
C37	0.0847 (19)	0.0614 (15)	0.0661 (19)	0.0085 (14)	0.0052 (14)	0.0117 (13)
C38	0.100 (2)	0.0547 (14)	0.089 (2)	−0.0185 (15)	0.0223 (17)	−0.0017 (14)
C39	0.0652 (16)	0.0664 (15)	0.0586 (17)	0.0062 (13)	−0.0038 (12)	−0.0064 (13)
C40	0.112 (3)	0.143 (3)	0.092 (3)	−0.027 (3)	−0.007 (2)	−0.048 (2)
C41	0.116 (3)	0.148 (3)	0.099 (3)	0.054 (3)	0.000 (2)	−0.044 (3)
C42	0.229 (5)	0.100 (3)	0.065 (2)	−0.021 (3)	−0.008 (3)	−0.002 (2)

### Geometric parameters (Å, °)

**Table:**

P1—O2	1.4559 (16)	C21—C24	1.516 (5)
P1—O4	1.5655 (16)	C21—C23	1.543 (6)
P1—O3	1.5712 (15)	C21—C23A	1.559 (10)
P1—O1	1.5713 (17)	C22—H22A	0.96
O1—C4	1.417 (3)	C22—H22B	0.96
O3—C15	1.421 (2)	C22—H22C	0.96
O4—C29	1.434 (3)	C23—H23A	0.96
C1—C2	1.372 (3)	C23—H23B	0.96
C1—C6	1.401 (3)	C23—H23C	0.96
C1—C7	1.540 (3)	C24—H24A	0.96
C2—C3	1.384 (3)	C24—H24B	0.96
C2—H2	0.93	C24—H24C	0.96
C3—C4	1.382 (3)	C22A—H22D	0.96
C3—H3	0.93	C22A—H22E	0.96
C4—C5	1.396 (3)	C22A—H22F	0.96
C5—C6	1.400 (3)	C23A—H23D	0.96
C5—C11	1.548 (3)	C23A—H23E	0.96
C6—H6	0.93	C23A—H23F	0.96
C7—C8A	1.475 (9)	C24A—H24D	0.96
C7—C10	1.506 (9)	C24A—H24E	0.96
C7—C9A	1.517 (8)	C24A—H24F	0.96
C7—C10A	1.538 (9)	C25—C27	1.533 (4)
C7—C8	1.540 (8)	C25—C26	1.540 (3)
C7—C9	1.546 (7)	C25—C28	1.548 (4)
C8—H8A	0.96	C26—H26A	0.96
C8—H8B	0.96	C26—H26B	0.96
C8—H8C	0.96	C26—H26C	0.96
C9—H9A	0.96	C27—H27A	0.96
C9—H9B	0.96	C27—H27B	0.96
C9—H9C	0.96	C27—H27C	0.96
C10—H10A	0.96	C28—H28A	0.96
C10—H10B	0.96	C28—H28B	0.96
C10—H10C	0.96	C28—H28C	0.96
C8A—H8AA	0.96	C29—C30	1.380 (3)
C8A—H8AB	0.96	C29—C34	1.403 (3)
C8A—H8AC	0.96	C30—C31	1.387 (3)
C9A—H9AA	0.96	C30—H30	0.9300
C9A—H9AB	0.96	C31—C32	1.385 (3)
C9A—H9AC	0.96	C31—H31	0.9300
C10A—H10D	0.96	C32—C33	1.400 (3)
C10A—H10E	0.96	C32—C39	1.541 (3)
C10A—H10F	0.96	C33—C34	1.395 (3)
C11—C12	1.527 (4)	C33—H33	0.9300
C11—C14	1.528 (4)	C34—C35	1.547 (3)
C11—C13	1.533 (4)	C35—C36	1.538 (3)
C12—H12A	0.96	C35—C37	1.538 (4)
C12—H12B	0.96	C35—C38	1.542 (3)
C12—H12C	0.96	C36—H36A	0.96
C13—H13A	0.96	C36—H36B	0.96
C13—H13B	0.96	C36—H36C	0.96
C13—H13C	0.96	C37—H37A	0.96
C14—H14A	0.96	C37—H37B	0.96
C14—H14B	0.96	C37—H37C	0.96
C14—H14C	0.96	C38—H38A	0.96
C15—C16	1.376 (3)	C38—H38B	0.96
C15—C20	1.399 (3)	C38—H38C	0.96
C16—C17	1.383 (3)	C39—C42	1.494 (4)
C16—H16	0.9300	C39—C41	1.517 (4)
C17—C18	1.380 (3)	C39—C40	1.530 (4)
C17—H17	0.9300	C40—H40A	0.96
C18—C19	1.399 (3)	C40—H40B	0.96
C18—C21	1.542 (3)	C40—H40C	0.96
C19—C20	1.401 (3)	C41—H41A	0.96
C19—H19	0.9300	C41—H41B	0.96
C20—C25	1.545 (3)	C41—H41C	0.96
C21—C22A	1.472 (9)	C42—H42A	0.96
C21—C24A	1.483 (10)	C42—H42B	0.96
C21—C22	1.509 (5)	C42—H42C	0.96
			
O2—P1—O4	116.48 (9)	C22—C21—C23	107.2 (4)
O2—P1—O3	116.59 (9)	C24—C21—C23	107.4 (5)
O4—P1—O3	102.13 (8)	C18—C21—C23	109.8 (3)
O2—P1—O1	117.14 (9)	C22A—C21—C23A	109.6 (8)
O4—P1—O1	101.49 (9)	C24A—C21—C23A	107.6 (9)
O3—P1—O1	100.36 (9)	C22—C21—C23A	63.8 (6)
C4—O1—P1	129.05 (14)	C24—C21—C23A	51.8 (6)
C15—O3—P1	127.04 (13)	C18—C21—C23A	106.6 (4)
C29—O4—P1	129.11 (14)	C23—C21—C23A	143.2 (5)
C2—C1—C6	116.9 (2)	C21—C22—H22A	109.5
C2—C1—C7	122.5 (2)	C21—C22—H22B	109.5
C6—C1—C7	120.6 (2)	H22A—C22—H22B	109.5
C1—C2—C3	121.5 (2)	C21—C22—H22C	109.5
C1—C2—H2	119.3	H22A—C22—H22C	109.5
C3—C2—H2	119.3	H22B—C22—H22C	109.5
C4—C3—C2	119.7 (2)	C21—C23—H23A	109.5
C4—C3—H3	120.1	C21—C23—H23B	109.5
C2—C3—H3	120.1	H23A—C23—H23B	109.5
C3—C4—C5	122.4 (2)	C21—C23—H23C	109.5
C3—C4—O1	120.2 (2)	H23A—C23—H23C	109.5
C5—C4—O1	117.33 (18)	H23B—C23—H23C	109.5
C4—C5—C6	114.98 (19)	C21—C24—H24A	109.5
C4—C5—C11	123.2 (2)	C21—C24—H24B	109.5
C6—C5—C11	121.8 (2)	H24A—C24—H24B	109.5
C5—C6—C1	124.5 (2)	C21—C24—H24C	109.5
C5—C6—H6	117.8	H24A—C24—H24C	109.5
C1—C6—H6	117.8	H24B—C24—H24C	109.5
C8A—C7—C10	125.6 (7)	C21—C22A—H22D	109.5
C8A—C7—C9A	114.5 (7)	C21—C22A—H22E	109.5
C10—C7—C9A	79.3 (6)	H22D—C22A—H22E	109.5
C8A—C7—C10A	108.0 (8)	C21—C22A—H22F	109.5
C9A—C7—C10A	103.0 (6)	H22D—C22A—H22F	109.5
C10—C7—C8	108.6 (6)	H22E—C22A—H22F	109.5
C9A—C7—C8	135.6 (6)	C21—C23A—H23D	109.5
C10A—C7—C8	86.4 (6)	C21—C23A—H23E	109.5
C8A—C7—C1	112.0 (6)	H23D—C23A—H23E	109.5
C10—C7—C1	111.4 (5)	C21—C23A—H23F	109.5
C9A—C7—C1	109.2 (4)	H23D—C23A—H23F	109.5
C10A—C7—C1	109.7 (5)	H23E—C23A—H23F	109.5
C8—C7—C1	107.9 (4)	C21—C24A—H24D	109.5
C8A—C7—C9	79.7 (6)	C21—C24A—H24E	109.5
C10—C7—C9	112.2 (5)	H24D—C24A—H24E	109.5
C10A—C7—C9	130.9 (6)	C21—C24A—H24F	109.5
C8—C7—C9	104.7 (5)	H24D—C24A—H24F	109.5
C1—C7—C9	111.6 (3)	H24E—C24A—H24F	109.5
C7—C8—H8A	109.5	C27—C25—C26	108.3 (2)
C7—C8—H8B	109.5	C27—C25—C20	110.3 (2)
H8A—C8—H8B	109.5	C26—C25—C20	111.4 (2)
C7—C8—H8C	109.5	C27—C25—C28	110.4 (2)
H8A—C8—H8C	109.5	C26—C25—C28	106.5 (2)
H8B—C8—H8C	109.5	C20—C25—C28	109.8 (2)
C7—C9—H9A	109.5	C25—C26—H26A	109.5
C7—C9—H9B	109.5	C25—C26—H26B	109.5
H9A—C9—H9B	109.5	H26A—C26—H26B	109.5
C7—C9—H9C	109.5	C25—C26—H26C	109.5
H9A—C9—H9C	109.5	H26A—C26—H26C	109.5
H9B—C9—H9C	109.5	H26B—C26—H26C	109.5
C7—C10—H10A	109.5	C25—C27—H27A	109.5
C7—C10—H10B	109.5	C25—C27—H27B	109.5
H10A—C10—H10B	109.5	H27A—C27—H27B	109.5
C7—C10—H10C	109.5	C25—C27—H27C	109.5
H10A—C10—H10C	109.5	H27A—C27—H27C	109.5
H10B—C10—H10C	109.5	H27B—C27—H27C	109.5
C7—C8A—H8AA	109.5	C25—C28—H28A	109.5
C7—C8A—H8AB	109.5	C25—C28—H28B	109.5
H8AA—C8A—H8AB	109.5	H28A—C28—H28B	109.5
C7—C8A—H8AC	109.5	C25—C28—H28C	109.5
H8AA—C8A—H8AC	109.5	H28A—C28—H28C	109.5
H8AB—C8A—H8AC	109.5	H28B—C28—H28C	109.5
C7—C9A—H9AA	109.5	C30—C29—C34	122.4 (2)
C7—C9A—H9AB	109.5	C30—C29—O4	120.71 (19)
H9AA—C9A—H9AB	109.5	C34—C29—O4	116.79 (19)
C7—C9A—H9AC	109.5	C29—C30—C31	119.6 (2)
H9AA—C9A—H9AC	109.5	C29—C30—H30	120.2
H9AB—C9A—H9AC	109.5	C31—C30—H30	120.2
C7—C10A—H10D	109.5	C32—C31—C30	121.6 (2)
C7—C10A—H10E	109.5	C32—C31—H31	119.2
H10D—C10A—H10E	109.5	C30—C31—H31	119.2
C7—C10A—H10F	109.5	C31—C32—C33	116.3 (2)
H10D—C10A—H10F	109.5	C31—C32—C39	122.2 (2)
H10E—C10A—H10F	109.5	C33—C32—C39	121.5 (2)
C12—C11—C14	108.6 (3)	C34—C33—C32	125.1 (2)
C12—C11—C13	109.9 (2)	C34—C33—H33	117.4
C14—C11—C13	106.4 (2)	C32—C33—H33	117.4
C12—C11—C5	110.4 (2)	C33—C34—C29	114.9 (2)
C14—C11—C5	111.6 (2)	C33—C34—C35	122.29 (19)
C13—C11—C5	109.8 (2)	C29—C34—C35	122.8 (2)
C11—C12—H12A	109.5	C36—C35—C37	110.7 (2)
C11—C12—H12B	109.5	C36—C35—C38	106.7 (2)
H12A—C12—H12B	109.5	C37—C35—C38	107.8 (2)
C11—C12—H12C	109.5	C36—C35—C34	111.00 (19)
H12A—C12—H12C	109.5	C37—C35—C34	109.43 (19)
H12B—C12—H12C	109.5	C38—C35—C34	111.2 (2)
C11—C13—H13A	109.5	C35—C36—H36A	109.5
C11—C13—H13B	109.5	C35—C36—H36B	109.5
H13A—C13—H13B	109.5	H36A—C36—H36B	109.5
C11—C13—H13C	109.5	C35—C36—H36C	109.5
H13A—C13—H13C	109.5	H36A—C36—H36C	109.5
H13B—C13—H13C	109.5	H36B—C36—H36C	109.5
C11—C14—H14A	109.5	C35—C37—H37A	109.5
C11—C14—H14B	109.5	C35—C37—H37B	109.5
H14A—C14—H14B	109.5	H37A—C37—H37B	109.5
C11—C14—H14C	109.5	C35—C37—H37C	109.5
H14A—C14—H14C	109.5	H37A—C37—H37C	109.5
H14B—C14—H14C	109.5	H37B—C37—H37C	109.5
C16—C15—C20	121.93 (19)	C35—C38—H38A	109.5
C16—C15—O3	120.82 (19)	C35—C38—H38B	109.5
C20—C15—O3	117.23 (18)	H38A—C38—H38B	109.5
C15—C16—C17	120.4 (2)	C35—C38—H38C	109.5
C15—C16—H16	119.8	H38A—C38—H38C	109.5
C17—C16—H16	119.8	H38B—C38—H38C	109.5
C18—C17—C16	121.1 (2)	C42—C39—C41	111.2 (3)
C18—C17—H17	119.4	C42—C39—C40	106.9 (3)
C16—C17—H17	119.4	C41—C39—C40	105.7 (3)
C17—C18—C19	116.6 (2)	C42—C39—C32	113.2 (2)
C17—C18—C21	121.9 (2)	C41—C39—C32	108.2 (2)
C19—C18—C21	121.5 (2)	C40—C39—C32	111.4 (2)
C18—C19—C20	124.7 (2)	C39—C40—H40A	109.5
C18—C19—H19	117.7	C39—C40—H40B	109.5
C20—C19—H19	117.7	H40A—C40—H40B	109.5
C15—C20—C19	115.04 (19)	C39—C40—H40C	109.5
C15—C20—C25	122.98 (19)	H40A—C40—H40C	109.5
C19—C20—C25	121.98 (19)	H40B—C40—H40C	109.5
C22A—C21—C24A	112.8 (9)	C39—C41—H41A	109.5
C22A—C21—C22	140.6 (5)	C39—C41—H41B	109.5
C24A—C21—C22	46.0 (7)	H41A—C41—H41B	109.5
C22A—C21—C24	58.8 (7)	C39—C41—H41C	109.5
C24A—C21—C24	135.4 (6)	H41A—C41—H41C	109.5
C22—C21—C24	109.5 (4)	H41B—C41—H41C	109.5
C22A—C21—C18	108.6 (5)	C39—C42—H42A	109.5
C24A—C21—C18	111.5 (5)	C39—C42—H42B	109.5
C22—C21—C18	110.5 (3)	H42A—C42—H42B	109.5
C24—C21—C18	112.4 (3)	C39—C42—H42C	109.5
C22A—C21—C23	53.0 (6)	H42A—C42—H42C	109.5
C24A—C21—C23	63.5 (8)	H42B—C42—H42C	109.5
			
O2—P1—O1—C4	36.4 (2)	C16—C15—C20—C19	−4.7 (3)
O4—P1—O1—C4	−91.55 (18)	O3—C15—C20—C19	173.3 (2)
O3—P1—O1—C4	163.67 (17)	C16—C15—C20—C25	175.0 (2)
O2—P1—O3—C15	39.9 (2)	O3—C15—C20—C25	−6.9 (3)
O4—P1—O3—C15	168.08 (18)	C18—C19—C20—C15	1.8 (4)
O1—P1—O3—C15	−87.66 (19)	C18—C19—C20—C25	−178.0 (2)
O2—P1—O4—C29	35.9 (2)	C17—C18—C21—C22A	−72.9 (8)
O3—P1—O4—C29	−92.31 (18)	C19—C18—C21—C22A	107.2 (8)
O1—P1—O4—C29	164.32 (17)	C17—C18—C21—C24A	162.2 (9)
C6—C1—C2—C3	−0.1 (3)	C19—C18—C21—C24A	−17.6 (9)
C7—C1—C2—C3	179.4 (2)	C17—C18—C21—C22	112.7 (4)
C1—C2—C3—C4	−0.5 (3)	C19—C18—C21—C22	−67.1 (4)
C2—C3—C4—C5	1.5 (3)	C17—C18—C21—C24	−9.9 (6)
C2—C3—C4—O1	−175.8 (2)	C19—C18—C21—C24	170.3 (5)
P1—O1—C4—C3	−15.8 (3)	C17—C18—C21—C23	−129.3 (4)
P1—O1—C4—C5	166.79 (16)	C19—C18—C21—C23	50.9 (5)
C3—C4—C5—C6	−1.8 (3)	C17—C18—C21—C23A	45.0 (7)
O1—C4—C5—C6	175.57 (18)	C19—C18—C21—C23A	−134.8 (7)
C3—C4—C5—C11	177.9 (2)	C15—C20—C25—C27	65.6 (3)
O1—C4—C5—C11	−4.7 (3)	C19—C20—C25—C27	−114.6 (3)
C4—C5—C6—C1	1.2 (3)	C15—C20—C25—C26	−174.0 (2)
C11—C5—C6—C1	−178.5 (2)	C19—C20—C25—C26	5.8 (4)
C2—C1—C6—C5	−0.3 (3)	C15—C20—C25—C28	−56.2 (3)
C7—C1—C6—C5	−179.8 (2)	C19—C20—C25—C28	123.5 (2)
C2—C1—C7—C8A	−96.1 (8)	P1—O4—C29—C30	−18.0 (3)
C6—C1—C7—C8A	83.3 (8)	P1—O4—C29—C34	165.17 (15)
C2—C1—C7—C10	117.5 (6)	C34—C29—C30—C31	0.0 (3)
C6—C1—C7—C10	−63.0 (6)	O4—C29—C30—C31	−176.6 (2)
C2—C1—C7—C9A	31.8 (8)	C29—C30—C31—C32	1.0 (4)
C6—C1—C7—C9A	−148.8 (8)	C30—C31—C32—C33	−1.0 (3)
C2—C1—C7—C10A	144.0 (7)	C30—C31—C32—C39	177.0 (2)
C6—C1—C7—C10A	−36.6 (7)	C31—C32—C33—C34	−0.1 (3)
C2—C1—C7—C8	−123.4 (5)	C39—C32—C33—C34	−178.1 (2)
C6—C1—C7—C8	56.1 (5)	C32—C33—C34—C29	1.0 (3)
C2—C1—C7—C9	−8.8 (7)	C32—C33—C34—C35	−180.0 (2)
C6—C1—C7—C9	170.6 (6)	C30—C29—C34—C33	−1.0 (3)
C4—C5—C11—C12	63.7 (3)	O4—C29—C34—C33	175.77 (18)
C6—C5—C11—C12	−116.6 (3)	C30—C29—C34—C35	−180.0 (2)
C4—C5—C11—C14	−175.4 (3)	O4—C29—C34—C35	−3.2 (3)
C6—C5—C11—C14	4.3 (4)	C33—C34—C35—C36	124.4 (2)
C4—C5—C11—C13	−57.7 (3)	C29—C34—C35—C36	−56.7 (3)
C6—C5—C11—C13	122.0 (3)	C33—C34—C35—C37	−113.1 (2)
P1—O3—C15—C16	−3.4 (3)	C29—C34—C35—C37	65.8 (3)
P1—O3—C15—C20	178.51 (16)	C33—C34—C35—C38	5.8 (3)
C20—C15—C16—C17	3.6 (4)	C29—C34—C35—C38	−175.3 (2)
O3—C15—C16—C17	−174.4 (2)	C31—C32—C39—C42	17.5 (4)
C15—C16—C17—C18	0.9 (4)	C33—C32—C39—C42	−164.6 (3)
C16—C17—C18—C19	−3.6 (4)	C31—C32—C39—C41	−106.1 (3)
C16—C17—C18—C21	176.5 (2)	C33—C32—C39—C41	71.7 (3)
C17—C18—C19—C20	2.3 (4)	C31—C32—C39—C40	138.1 (3)
C21—C18—C19—C20	−177.9 (2)	C33—C32—C39—C40	−44.0 (3)

### Hydrogen-bond geometry (Å, °)

**Table:**

*D*—H···*A*	*D*—H	H···*A*	*D*···*A*	*D*—H···*A*
C3—H3···O2	0.93	2.32	3.017 (3)	132
C12—H12C···O1	0.96	2.36	3.022 (4)	125
C13—H13A···O1	0.96	2.37	2.996 (3)	122
C16—H16···O2	0.93	2.38	3.023 (3)	126
C27—H27B···O3	0.96	2.40	3.032 (3)	123
C28—H28B···O3	0.96	2.33	2.990 (4)	125
C30—H30···O2	0.93	2.32	3.019 (3)	132
C36—H36A···O4	0.96	2.31	2.969 (3)	125
C37—H37C···O4	0.96	2.40	3.044 (3)	124
